# The Mid-Term Effect of Preservative-Free Artificial Tears Containing Hyaluronic Acid on Dry Eye Incidence after Cataract Surgery: A Retrospective Cohort Study

**DOI:** 10.3390/diagnostics14171848

**Published:** 2024-08-24

**Authors:** Chia-Yi Lee, Shun-Fa Yang, Hung-Chi Chen, Ie-Bin Lian, Chin-Te Huang, Jing-Yang Huang, Chao-Kai Chang

**Affiliations:** 1 Institute of Medicine, Chung Shan Medical University, Taichung 40201, Taiwan; 2 Nobel Eye Institute, Taipei 10041, Taiwan; 3 Department of Ophthalmology, Jen-Ai Hospital Dali Branch, Taichung 41265, Taiwan; 4 Department of Medical Research, Chung Shan Medical University Hospital, Taichung 40201, Taiwan; 5 Department of Ophthalmology, Chang Gung Memorial Hospital, Linkou, Taoyuan 33305, Taiwan; 6 Center for Tissue Engineering, Chang Gung Memorial Hospital, Linkou, Taoyuan 33305, Taiwan; 7 Department of Medicine, Chang Gung University College of Medicine, Taoyuan 33305, Taiwan; 8 Institute of Statistical and Information Science, National Changhua University of Education, Chunghua 50007, Taiwan; 9 Department of Ophthalmology, Chung Shan Medical University Hospital, Taichung 40201, Taiwan; 10 Department of Ophthalmology, School of Medicine, Chung Shan Medical University, Taichung 40201, Taiwan; 11 Department of Optometry, Da-Yeh University, Chunghua 51591, Taiwan

**Keywords:** hyaluronic acid, dry eye disease, cataract surgery, superficial keratitis, tear break-up time

## Abstract

The aim of this study is to survey the effectiveness of preservative-free artificial tears containing hyaluronic acid (HA) on post-cataract surgery dry eye disease (DED) prevention. A retrospective cohort study was performed, and patients that received cataract surgeries were divided into either an HA group or non-HA group depending on the artificial tear they used. A total of 37 and 74 eyes were enrolled into the HA and non-HA groups, respectively, after the selection. The primary outcomes are postoperative superficial keratitis and multiple (>3) DED symptoms. The generalized linear model was utilized to calculate the adjusted odds ratio (aOR) and 95% confidence interval (CI) of primary outcomes between the two groups. There were 10 and 2 episodes of superficial keratitis in the non-HA group and HA group, respectively, and the HA group demonstrated a significantly lower incidence of superficial keratitis (*p* < 0.001). Moreover, 13 and 5 patients developed multiple DED symptoms in the non-HA and HA groups, and the HA group illustrated fewer multiple DED symptoms (*p* = 0.024). The lower preoperative tear break-up time (TBUT) was correlated with superficial keratitis in the HA group (*p* = 0.043), while old age, low preoperative TBUT and ocular surface staining were associated with superficial keratitis in the non-HA group (all *p* < 0.05). Lower preoperative TBUT was correlated with multiple DED symptoms in the HA group (*p* = 0.020), while female sex, low preoperative TBUT and any DED symptoms were associated with multiple DED symptoms in the non-HA group (all *p* < 0.05). In conclusion, the usage of preservative-free artificial tears containing HA is associated with lower postoperative DED events.

## 1. Introduction

The cataract is an ophthalmic disease that contributes to visual impairment or blindness for more than 80 million humans [1]. The common symptoms of a formed cataract include progressively decreased vision, halos, monocular diplopia, a foggy sensation, and difficulty with near vision [1,2]. The only effective intervention to handle impaired visual acuity due to cataracts is cataract surgery [3]. In most cases, the postoperative visual acuity is elevated after cataract surgery [4,5], and several intraocular lens (IOL) with different functions can be utilized to yield a fair postoperative visual outcome [6,7].

Despite a smooth postoperative recovery in the majority of cases, complications could still occur after the cataract surgery [8]. The postoperative endophthalmitis is a serious complication after cataract surgery that can cause severe visual impairment, and eyeball removal is warranted in some cases [9]. In addition, persistent corneal edema or corneal decompensation can develop in patients that receive excessive phaco-power intraoperatively or those with a previously impaired corneal endothelium [10]. In addition, postoperative dry eye disease (DED) after cataract surgery is estimated to develop in more than 30 percent of cases [11]. Although severe visual impairment was rarely reported after postoperative DED, postoperative DED can cause significant visual disturbance and corneal injury [12,13]. Accordingly, prevention measures for postoperative DED should be implements.

Artificial tears have been administered for both general DED and postoperative DED for decades [14,15]. The components of artificial tears in different products are varied [12], and artificial tears containing hyaluronic acid (HA) have gained popularity recently due to their effectiveness against DED signs and symptoms [13,16]. Some studies have compared the effectiveness of preservative-free artificial tears containing HA and other preservative-free components on DED after cataract surgery, revealing fair outcomes [11,17,18]. However, few studies have directly compared preservative-free artificial tears containing HA versus other preservative-free artificial tears. Moreover, the duration of post-cataract surgery DED can persist for 6 weeks [19], while previous studies only had follow-up periods of around one month [11,17,18]. Also, whether the factors for poor therapeutic outcomes of preservative-free artificial tears containing HA are the same as the general risk of DED needs to be elucidated.

Accordingly, the purpose of our study is to evaluate the mid-term effectiveness of one preservative-free artificial tear containing HA on managing DED-related signs and symptoms after cataract surgery. Other risk factors for postoperative DED were also analyzed.

## 2. Materials and Methods

### 2.1. Participant Selection

A retrospective cohort study was performed at the Nobel Eye Institute, which is a medical institution that has several clinics throughout Taiwan. The participants were selected into the present study if (1) they were between 50 and 100 years of age, (2) a complicated or senile cataract was found at the Nobel Eye Institute, (3) cataract surgery and IOL implantation were performed at the Nobel Eye Institute, and (4) they had visited the Nobel Eye Institute for at least two months after cataract surgery. Moreover, the following exclusion criteria were adopted to exclude patients with prominent ocular morbidities: (1) preoperative corrected distance visual acuity (CDVA) lower than hand motion, (2) preceding eyeball rupture history, (3) preceding centrally involved corneal ulcer or central corneal opacity, (4) preceding advanced retinal diseases including macula-involved retinal detachment or end-stage age-related macular degeneration, (5) neovascular glaucoma, (6) ischemic optic neuropathy or optic neuritis, and (7) the usage of more than one type of artificial tear during the two-month postoperative follow-up. Subsequently, the patients were divided into an HA group and non-HA group based on the artificial tears applied after cataract surgery. The patients in the HA group used one type of preservative-free artificial tear containing HA (Systane ^®^ Hydration UD, Alcon, Fort Worth, TX, USA) after cataract surgery, while the patients in the non-HA group used a non-HA preservative-free artificial tear (Systane ^®^ Ultra UD, Alcon, Fort Worth, TX, USA). The decision on whether or not to use artificial tears containing HA was made by the patients since the artificial tear product containing HA is self-paid in Taiwan. Only the first eye that underwent cataract surgery was included in our study, and one eye administered artificial tears containing HA was matched with two eyes that did not use artificial tears containing HA within a two-month period. After the whole selection, a total of 37 eyes from 37 patients were enrolled in the HA group, and 74 eyes from 74 patients were enrolled into the non-HA group. We chose to have unequal case numbers because we needed to achieve adequate statistical power and a 1:2 ratio was acceptable in our previous publications [20,21]. The statistical power of our study was 0.81 with a 0.05 alpha value, and the medium effect size was generated via G*power version 3.1.9.2 (Heinrich Heine Universität at Düsseldorf, Germany).

### 2.2. Cataract Surgery

All the cataract surgeries were performed by one experienced cataract specialist (C.-Y.L.), and one phacoemulsification device (Quatera, Carl Zeiss, Göschwitzer Str., Jena, Germany) was used for the cataract surgeries. The main corneal incision was performed using a superior approach, and an ophthalmic viscoelastic device was infused into the anterior chamber. After completing the continuous curvilinear capsulorhexis, hydrodissection was performed, followed by side-port creation. The phaco-chop technique was administrated to clean the lens nucleus, and the cortex material was extracted using an infusion–aspiration probe. The IOL was put into the capsular bag and the residual ophthalmic viscoelastic device was removed from the eye. Finally, the hydro-seal technique was applied to close the main incision as well as the side-port. After the completion of surgery, a 0.5% moxifloxacin eyedrop and 0.3% tobramycin/0.1% dexamethasone ointment were introduced to the corneal surface. Postoperatively, 1% prednisolone solution was administered four times a day, 0.5% levofloxacin solution four times a day, and 0.3% tobramycin/0.1% dexamethasone ointment was used before sleeping for the first 7 days. After that, the combined 0.1% dexamethasone/0.35% neomycin eyedrop administered four times a day was applied for another 7 days. The 4% sulfamethoxazole and 0.02% fluorometholone solutions were applied two times a day for three weeks. Regarding the usage of artificial tears, artificial tears both with and without HA were adopted for at least two months, and the patients could decide whether to use the artificial tears for more time.

### 2.3. Dry Eye Examinations

All the patients in our study received the same preoperative and postoperative exams. The preoperative exams involved uncorrected distance visual acuity (UDVA) and CDVA measurement, and cyclopegic refraction of both sphere power and cylinder power using an autorefractor (KR-8900, Topcon, Itabashi-ku, Tokyo, Japan). Also, the tear break-up time (TBUT), Schirmer II test, and ocular surface staining by fluorescein were performed for all the patients, and whether the patients had previous DED symptoms including dryness, foreign body sensation, grittiness, burning sensations, soreness, itching, photophobia, discharge, or redness was recorded in the medical document. The TBUT refers to fluorescein-stained TBUT, and superficial keratitis refers to the presence of punctate corneal epithelial damage, as decided by ocular surface staining. To obtain more detail from ocular surface staining, fluorescein dye was put onto the inferior bulbar conjunctival surface. Subsequently, the individuals were requested to blink the eye three times, and photos of the ocular surface, involving the cornea and conjunctiva, were captured via the accessory camera of the slit-lamp microscope. The epithelial defect of cornea was scored from 0 (absence) to 5 (diffuse punctate keratitis in the entire cornea) based on the Oxford Scheme [22]. Most items of the DED symptoms we evaluated were referenced to the ocular surface disease index [23]. The routine postoperative exams of cataract surgery also involved the UDVA, IOP, manifest sphere power and cylinder power. Ocular surface staining by fluorescein for superficial keratitis and the subjective symptoms were also evaluated following cataract surgery. Of note, the postoperative exams employed identical devices during the preoperative exams. The ophthalmic exams were carried out before, one day after, one week after, one month after, and two months after the cataract surgery in our study. The spherical equivalent (SE) in our study was regarded as the sphere power plus half the cylinder power.

### 2.4. Statistical Analysis

SPSS version 20.0 (SPSS Inc., Chicago, IL, USA) was adapted for the statistical analysis in our study. The Shapiro–Wilk test was adapted to check the normality of our data, and the results presented the abnormal distribution of all data in our study (all *p* < 0.05). The descriptive analysis was adapted to reveal the age, sex, pre-existing systemic and ophthalmic diseases, preceding surgeries, UDVA, CDVA, preoperative refraction, Schirmer II test, superficial keratitis, numbers of DED-related symptoms, and postoperative outcomes in both groups. The Mann–Whitney U test and Fisher’s exact test were adapted to evaluate the preoperative and postoperative features between the HA group and non-HA group depending on the property of each parameter. In the next step, the generalized linear model was adapted to investigate the incidence of postoperative superficial keratitis and multiple DED symptoms (more than or equal to three DED-related symptoms) between the HA and non-HA groups after the adjustment of age and sex. The adjusted odds ratio (aOR) with a 95% confidence interval (CI) for superficial keratitis and multiple DED-related symptoms between the two groups was yielded via the generalized linear model. Moreover, the generalized linear model was adapted to survey the potential risk factors for postoperative superficial keratitis and multiple DED-related symptoms in the HA group and non-HA group separately. The potential risk factors applied in the analysis include old age (more than 70 years old), female sex, low preoperative TBUT (<10 s), low preoperative Schirmer II test (<10 mm), positive ocular surface staining, and the presence of any DED-related symptom. A *p* value less than 0.05 indicated statistical significance in our study, and a *p* value less than 0.001 was revealed as *p* < 0.001.

## 3. Results

The baseline characteristics of the two groups are presented in Table 1. The mean age was 61.25 ± 10.32 years in the non-HA group and 60.70 ± 10.87 years in the HA group, which showed no significant difference (*p* = 0.733). Also, the distributions of sex, laterality, previous systemic and ophthalmic diseases and preceding ocular surgeries did not show any significant difference (all *p* > 0.05). Regarding the preoperative ocular and DED-related parameters, all the items demonstrated insignificant differences between the HA group and non-HA group (all *p* > 0.05) (Table 1).

The initial postoperative UDVA was 0.10 ± 0.11 and 0.09 ± 0.15 in the non-HA and HA groups, respectively, without a significant difference (*p* = 0.641). Two months postoperatively, however, the HA group presented a significantly better UDVA than the non-HA group (0.07 ± 0.09 versus 0.03 ± 0.07, *p* = 0.018). On the other hand, the SE did not reveal any significant difference between the two groups throughout the study period (all *p* > 0.05) (Table 2). There were 10 and 2 episodes of superficial keratitis in the non-HA group and HA group, respectively. Regarding classifications of corneal staining by Oxford scale of our patients, the two superficial keratitis episodes in the HA group were grade 1, while there were five, four and one superficial keratitis episodes belonging to grade 1, 2 and 3 in the non-HA group. After adjusting the effect of age and sex, the HA group demonstrated a significantly lower incidence of postoperative superficial keratitis compared to the non-HA group (aOR: 0.577, 95% CI: 0.329–0.868, *p* < 0.001) (Table 3). Regarding the DED symptoms, 2 and 0, 14 and 6, and 15 and 5 eyes developed multiple DED symptoms in the non-HA group and HA group at one day, one week and one month postoperatively. Finally, 13 and 5 eyes developed multiple DED symptoms in the non-HA and HA groups, respectively, and the HA group demonstrated a lower risk of multiple DED symptoms after cataract surgery (aOR: 0.833, 95% CI: 0.706–0.959, *p* = 0.024) (Table 3).

Concerning the risk factors for superficial keratitis, a lower preoperative TBUT was correlated with postoperative superficial keratitis in the HA group (*p* = 0.043), while old age, low preoperative TBUT and ocular surface staining were associated with postoperative superficial keratitis in the non-HA group (all *p* < 0.05) (Table 4). Regarding the risk factors for multiple DED symptoms, a lower preoperative TBUT was correlated with multiple postoperative DED symptoms in the HA group (*p* = 0.020), while the female sex, low preoperative TBUT and any DED symptoms were associated with multiple postoperative DED symptoms in the non-HA group (all *p* < 0.05) (Table 5).

## 4. Discussion

In short, our study demonstrated the lower incidence of postoperative superficial keratitis and DED-related symptoms in patients that received preservative-free artificial tears containing HA. Moreover, the postoperative UDVA was significantly better in the HA group compared to in the non-HA group. Additionally, the non-HA group was associated with more risk factors for postoperative DED compared to the HA group.

DED is a multifactorial disease [16], and tear film instability and loss of homeostasis on the ocular surface are the fundamental components for DED [24]. Tear film instability causes damage to the corneal epithelium and the release of several cytokines; osmolarity of the tear film then increases, and the stability of tear film decreases [25]. This is the vicious cycle of DED [25]. Inflammatory cytokines serve an important role in the development of DED, as interleukin expression increases in patients with this condition [26]. Moreover, interferon and tumor necrosis factor are also cytokines that become elevated in the formation of DED [27]. Oxidative stress is another pathophysiology for the development of DED [28]. High oxidative stress causes DNA damage and lipid peroxidation, which contributes to the subsequent development of DED [29]. Clinically, antioxidant levels significantly decrease in patients with pre-existing DED and those who have undergone keratorefractive surgery [30]. On the other hand, ocular surface damage could also lead to the development of DED [31]. A damaged corneal epithelium is correlated with lower tear film stability and subjective symptoms [32], and both of them are components for DED [16]. Impaired goblet cells resulting from chemical burns or autoimmune disease reduce mucin secretion, leading to subsequent DED [33]. HA is a long-chain structure shown to have anti-inflammatory and wound-healing functions in previous research [34]. The interleukin level decreases after the application of HA and epigallocatechin gallate [35]. In addition, HA can serve as a free radical scavenger, where its large molecules can attenuate oxidative stress [36]. Moreover, the usage of HA can accelerate corneal re-epithelialization, as shown in an experimental study on mouse corneas [16]. Since HA has several characteristics that slow DED progression [16], we speculate that the application of preservative-free artificial tears containing HA could reduce the incidence of postoperative DED compared to other preservative-free artificial tears. This hypothesis was supported by the results of this study.

In our study, the incidence of postoperative superficial keratitis and DED symptoms were significantly lower in patients that used artificial tears containing HA than those took artificial tears that did not contain HA. In previous research, artificial tears containing HA could effectively slow DED development compared to general artificial tears [37]. In addition, subjective DED symptoms demonstrated a significant reduction after refractive surgery with the application of artificial tears containing HA [38]. Regarding the correlation of post-cataract surgery DED and artificial tears containing HA, two previous studies demonstrated significant improvements in both DED-related signs and symptoms after the usage of artificial tears containing HA [17,18]. Another earlier study also illustrated that HA/trehalose ophthalmic solution effectively reduces post-cataract surgery DED signs and symptoms for approximately one month [11]. Still, whether the effectiveness of preservative-free artificial tears containing HA is different from other preservative-free artificial tears in the mid-term period remains unclear. To our knowledge, our findings may represent preliminary evidence of the mid-term effectiveness of artificial tears containing HA in managing postoperative DED after cataract surgery. In addition, the non-HA group applied preservative-free artificial tears after cataract surgery; thus, the influence of preservative agents on the development of DED could be minimal in our study. Furthermore, the age and sex were adjusted in the generalized linear model, and both of them are significant risk factors for DED occurrence [24]. There was no prominent intraoperative complication (posterior capsular rupture, iris dialysis, zonular dialysis or lens drops) in any of the eyes in our study. Also, the duration of each cataract surgery in our study ranged from 13 to 22 min, which may not significantly impact postoperative DED. Moreover, four eyes were diagnosed with age-related macular degeneration and one with anterior uveitis, while the conditions of these diseases were stable and no eye received related treatment during the last 3 years. As a consequence, the application of artificial tears containing HA may be an independent protective factor for the incidence of DED after cataract surgery. The cataract surgery causes ocular surface damage due to the main corneal incision and side-port creation [39], and the phacoemulsification procedure during cataract surgery increases oxidative stress in the eye [40]. Moreover, the high viscosity of artificial tears containing HA may smooth the contact between the ocular surface and eyelid; thus, less irritation was felt [41]. Thus, it is reasonable to conclude that the use of artificial tears containing HA is associated with better post-cataract surgery recovery and a lower incidence of DED signs and symptoms.

Concerning the risk factors for postoperative superficial keratitis, a low TBUT was associated with a higher incidence of superficial keratitis in both groups while old age and ocular surface staining was associated with a higher incidence of superficial keratitis in the non-HA group. In a previous study, the TBUT was used to assess tear film stability, with decreased TBUT serving as an important biomarker for the occurrence of DED [27,42]. The decreased TBUT in both groups was significantly correlated with the formation of postoperative superficial keratitis, which may indicate the crucial role of TBUT on the post-cataract surgery corneal condition. On the other hand, old age and pre-existing ocular surface staining have both been established as risk factors for DED in the literature [24]. However, these two parameters were only associated with postoperative superficial keratitis in the non-HA group, and not in the HA group. The possible explanation is that artificial tears containing HA provide better protection against postoperative DED; thus, only the most prominent factor (i.e., the low postoperative TBUT) affects the development of postoperative superficial keratitis in the HA group. Another possible explanation is that the incidence of postoperative superficial keratitis was relatively low in the HA group compared to in the non-HA group; thus, the statistical results may tend toward insignificance. Regarding postoperative DED symptoms, low preoperative TBUT is still associated with more DED symptoms in both groups, which indicates the universal effect of TBUT on postoperative DED. The female sex and preoperative DED symptoms were associated with postoperative DED symptoms in the non-HA group. The female sex is a prominent risk factor for DED, and females tend to experience more DED symptoms than males [24,43]. Also, pre-existing DED symptoms may affect the incidence of postoperative DED symptoms [44]. Similarly, these associations only occurred in the non-HA group, which may indicate the effectiveness of artificial tears containing HA on the DED symptoms after cataract surgery. Overall, the risk factors for poor therapeutic outcomes for the post-cataract surgery DED in our study are similar to the risk factors for DED in the previous literature [24,27,42,43,44]. Still, our results further demonstrated that patients using preservative-free artificial tears containing HA had fewer risk factors for poor DED outcomes (i.e., only low TBUT) compared to those using other types of preservative-free artificial tears. Consequently, preservative-free artificial tears containing HA may be suitable for patients with cataracts and pre-existing risk factors for DED.

Regarding the postoperative outcome in the HA and non-HA groups, the postoperative UDVAs were similar between the two groups until two months postoperatively in which the final UDVA was significantly better in the HA group than the non-HA group. Prominent DED after cataract surgery can influence the visual quality, overall satisfaction and quality of life of patients [11]. In addition, the presence of postoperative DED diminishes the visual outcome following keratorefractive surgery [45]. The lower incidence of postoperative DED in the HA group, according to the DED-related signs and symptoms, may have some benefits on postoperative visual acuity. Still, the absolute value of postoperative UDVA difference is not significant in clinical practice; thus, the influence of different artificial tears on postoperative vision may not be prominent. On the other hand, the postoperative SE showed similar values between the two groups during the whole follow-up interval. This phenomenon may indicate that the DED severity in both groups is acceptable and did not cause prominent refractive error, which is observed in severe DED [46].

For the visual and refractive results between our study and the previous research, the mean final postoperative UDVA in the HA group was 0.03. In the earlier literature, the mean postoperative UDVA was around 0.10; thus, the postoperative UDVA in our study may not be inferior [47]. When it comes to the refractive outcome, the mean final postoperative SEs were −0.25 D and −0.24 D in the non-HA group and HA group, respectively, which were near the minimal refraction measurement, −0.25 D, and might not have a significant effect on postoperative visual acuity. In the earlier literature that inserted a toric IOL, the mean postoperative SE was approximately −0.48 D, and the SEs two months postoperatively in our study are comparable to the preceding finding [48]. In addition, the mean postoperative SE in an article that inserted a multifocal IOL was approximately −0.60 D, and the postoperative refraction of the both groups in our study was compatible to those without a prominent preoperative astigmatism [49]. As a result, the visual and refractive outcomes in the non-HA and HA groups could be acceptable considering the results of previous articles, and the quality of cataract surgery in our institution may be adequate.

There are some limitations in our study. Firstly, the retrospective design of our study could have diminished the homogeneity of the study population, although no significant differences for any of the preoperative parameters were found between groups. Certain DED-related factors, including the exact intraoperative condition of cataract surgery, the duration of cataract surgery and the degree of pre-existing ophthalmic diseases, could not be fully controlled in our study due to its retrospective nature. Secondly, the total case numbers are relative inadequate with only 111 eyes enrolled in our study, which may have diminished the statistical power. In addition, several crucial DED-related evaluations including the ocular surface disease index questionnaire, Rose-Bengal staining, grading of puntate keratitis, postoperative TBUT, postoperative Schirmer II test and the frequency of DED symptoms in each individual were not arranged in our study due to its retrospective nature. Thus, the absence of systematic evaluations of DED may have reduced the integrity of our analysis. Moreover, the types of artificial tears were selected by the patients themselves, which may have introduced some subjective bias and influenced the results in terms of their self-reported symptoms. Finally, the patients did not apply artificial tears at the same frequency. Accordingly, the exact effectiveness of artificial tears containing HA on postoperative DED cannot be totally confirmed.

## 5. Conclusions

In conclusion, the application of preservative-free artificial tears containing HA is correlated with a lower risk of postoperative superficial and DED symptoms compared to other preservative-free artificial tears after cataract surgery. Furthermore, the number of predisposing factors for post-cataract surgery DED is also lower in patients that used preservative-free artificial tears containing HA. Consequently, the usage of preservative-free artificial tears containing HA may be recommended to those with pre-existing DED who are scheduled for cataract surgery. Further large-scale prospective research with randomization is necessary to confirm the results of our study and evaluate additional protocols for using preservative-free artificial tears containing HA to prevent post-cataract surgery DED.

## Figures and Tables

**Table 1:** The baseline features of the study population.

Feature	Non-HA Group (*N*: 74)	HA Group (*N*: 37)	*p*
Age (years, mean ± SD)	61.25 ± 10.32	60.70 ± 10.87	0.733
Sex (male: female)	34:40	21:16	0.318
Laterality (right: left)	29:45	15:22	0.891
Disease			0.854
Hypertension	4	2	
Diabetes mellitus	5	2	
Autoimmune disease	3	1	
Other	2	0	
Ophthalmic diseases			0.305
Retinal disease	2	2	
Uveitis	0	1	
Ocular surgery	6	2	0.717
UDVA (LogMAR)	0.45 ± 0.26	0.41 ± 0.28	0.409
CDVA (LogMAR)	0.34 ± 0.18	0.29 ± 0.17	0.146
Cycloplegia refraction (D)			
Sphere	−3.17 ± 4.56	−3.02 ± 5.41	0.858
Cylinder	−1.25 ± 0.73	−1.18 ± 0.82	0.617
SE	−3.79 ± 5.09	−3.61 ± 4.81	0.834
TBUT	12.15 ± 3.79	13.01 ± 3.57	0.213
Schirmer II test	15.76 ± 6.61	14.84 ± 7.24	0.486
Ocular surface stain	2	2	0.514
DED-related syndrome			0.803
0	52	24	
1	17	10	
2	5	3	
≥3	0	0	

CDVA: corrected distance visual acuity, D: diopter, DED: dry eye disease, HA: hyaluronic acid, *N*: number, SD: standard deviation, SE: spherical equivalent, TBUT: tear break-up time, UDVA: uncorrected distance visual acuity.

**Table 2:** Postoperative visual and refractive outcomes between the two groups.

Outcome	Non-HA Group (*N*: 74)	HA Group (*N*: 37)	*p*
UDVA			
1 day	0.10 ± 0.11	0.09 ± 0.15	0.641
1 week	0.08 ± 0.15	0.07 ± 0.11	0.688
1 month	0.08 ± 0.13	0.06 ± 0.08	0.364
2 months	0.07 ± 0.09	0.03 ± 0.07	0.018 *
SE			
1 day	−0.29 ± 0.31	−0.26 ± 0.34	0.615
1 week	−0.25 ± 0.28	−0.25 ± 0.30	0.996
1 month	−0.26 ± 0.24	−0.24 ± 0.23	0.637
2 months	−0.25 ± 0.25	−0.24 ± 0.21	0.808

HA: hyaluronic acid, *N*: number, SE: spherical equivalent, UDVA: uncorrected distance visual acuity. * denotes significant difference between groups.

**Table 3:** The incidence of postoperative superficial keratitis and multiple dry eye disease symptoms between the two groups.

DED Parameters	Non-HA Group (*N*: 74)	HA Group (*N*: 37)	*p*
Superficial keratitis			
Incidence	10	2	
Crude OR (95% CI)	Reference	0.415 (0.217–0.759)	<0.001 *
aOR (95% CI)	Reference	0.577 (0.329–0.868)	<0.001 *
Multiple DED symptoms			
Incidence	13	5	
Crude OR (95% CI)	Reference	0.719 (0.556–0.852)	0.002 *
aOR (95% CI)	Reference	0.833 (0.706–0.959)	0.024 *

aOR: adjusted odds ratio, CI: confidence interval, DED: dry eye disease, HA: hyaluronic acid, *N*: number. * denotes significant correlation to DED parameters.

**Table 4:** Risk factors for superficial keratitis in the two groups.

Factor	aOR	95% CI		*p*
		Lower	Upper	
HA group				
Old age	1.121	0.923	1.368	0.247
Female sex	1.065	0.872	1.395	0.394
Low preoperative TBUT	1.289	1.004	1.462	0.043 *
Low Preoperative Schirmer II test	1.117	0.902	1.287	0.176
Ocular surface stain	1.296	0.973	1.519	0.069
Any DED symptoms	1.077	0.899	1.317	0.348
Non-HA group				
Old age	1.245	1.098	1.466	0.033 *
Female sex	1.153	0.991	1.307	0.061
Low preoperative TBUT	1.376	1.138	1.551	0.016 *
Low Preoperative Schirmer II test	1.283	0.954	1.462	0.144
Ocular surface stain	1.323	1.149	1.576	0.018 *
Any DED symptoms	1.119	0.923	1.401	0.221

aOR: adjusted odds ratio, CI: confidence interval, DED: dry eye disease, HA: hyaluronic acid, TBUT: tear break-up time. * denotes significant correlation to superficial keratitis.

**Table 5:** Risk factors for multiple dry eye disease symptoms in the two groups.

Factor	aOR	95% CI		*p*
		Lower	Upper	
HA group				
Old age	1.005	0.822	1.257	0.748
Female sex	1.287	0.938	1.584	0.107
Low preoperative TBUT	1.394	1.021	1.627	0.020 *
Low Preoperative Schirmer II test	1.052	0.877	1.419	0.336
Ocular surface stain	1.096	0.913	1.255	0.531
Any DED symptoms	1.274	0.949	1.472	0.144
Non-HA group				
Old age	1.212	0.965	1.443	0.098
Female sex	1.299	1.018	1.485	0.035 *
Low preoperative TBUT	1.459	1.162	1.694	0.001 *
Low Preoperative Schirmer II test	1.229	0.867	1.453	0.245
Ocular surface stain	1.178	0.882	1.409	0.191
Any DED symptoms	1.297	1.056	1.565	0.027 *

aOR: adjusted odds ratio, CI: confidence interval, DED: dry eye disease, HA: hyaluronic acid, TBUT: tear break-up time. * denotes significant correlation to multiple DED symptoms.

## Data Availability

The original data used in this study are available from the corresponding author upon reasonable request.
